# Can microRNAs keep inflammasomes in check?

**DOI:** 10.3389/fgene.2013.00030

**Published:** 2013-03-13

**Authors:** Omar Qazi, Prasanna T. Parthasarathy, Richard Lockey, Narasaiah Kolliputi

**Affiliations:** Department of Internal Medicine, Division of Allergy and Immunology, Morsani College of Medicine, University of South FloridaTampa, FL, USA

Inflammasomes are multi-protein signaling platforms that are primed upon sensing pathogen-associated molecular patterns (PAMPs) as well as endogenous damage-associated molecular patterns (Kolliputi et al., 2010, 2012). Once primed, a complex forms between the inflammasome sensor, an adaptor protein with a caspase recruiter domain (ASC), and caspase-1, which leads to the cleavage of pro-inflammatory cytokines Interleukin-1beta and Interleukin-18 (IL-1B, IL-18). A specific inflammasome sensor, nucleoside-triphosphatase domain (NACHT), leucine rich repeat (LRR), and pyrin domain (PYD) domains-containing protein 3 (NLRP3) has garnered the attention of many secondary to its ability to function as a general sensor of cell stress, possessing a large number of endogenous and exogenous activators compared to other inflammasome sensors such as Interferon-inducible protein (AIM2) and NLR family CARD domain-containing protein 4 (NLRC4). This pathway has also been implicated in numerous diseases like gout, alzheimers, obesity, and diabetes (Heneka et al., 2013; Wei et al., 2013). Given the prevalent evidence that this pathway is involved in many medical conditions, current and further studies about inflammasome regulation could potentially allow pharmaceutical researchers to develop drugs for currently incurable diseases. The NLRP3 inflammasome complex can be regulated in three main ways; the degree of which the inflammasome complex is expressed, the amount of NLRP3 activators present and post-transcriptional modulation via microRNAs (miRNA). miRNAs are defined as a class of non-coding oligonucleotides which function in post-transcriptional regulation of gene expression (Bartel, 2009; Tamarapu et al., 2012). A particular miRNA may have multiple targets, and a given mRNA may have several miRNAs that may exhibit regulatory function on itself (Krek et al., 2005; Jalali et al., 2012). These oligonucleotide sequences have become a hot area of research as they are proving to become relevant in the pathogenesis and possible cure to multiple medical conditions (Holohan et al., 2012). In atherosclerosis it has been shown that elevated miR-155 levels are found in pro-inflammatory macrophages as well as in atherosclerotic lesions (Wei et al., 2013). In the September 2012 edition of the *Journal of Immunology*, Bauernfeind et al. (2012) present exciting findings on inflammasome sensor, NLRP3, by showing its negative regulation via a type of miRNA, miR-223. To investigate the potential role of post-transcriptional regulation on the inflammasome pathway, Bauernfeind et al. (2012) conducted several experiments in different cell lineages. Their initial study involved examining luciferase activity after transfection with a plasmid armed with luciferase on the 3′UTR of human NLRP3, as well as a genome wide miRNA precursor library in 293T cells. Of the miRNA precursor library, miR-223 showed the greatest promise, decreasing the activity of NLRP3. Bauernfeind et al. (2012) showed that miR-223 levels increase during granulopoiesis and are highest in mature neutrophils, while expression is absent in T and B cells. They also noted an inverse relationship with regards to miR-223 and NLRP3 during the maturation process in the monocyte lineage, showing a decrease in miR-223 levels, an increase in NLRP3 transcripts, and caspase activation after culturing with granulocyte-macrophage colony-stimulating factor (GM-CSF). This was also proven by Haneklaus et al. (2012) as they showed low NLRP3 protein levels in monocytes and significantly higher levels in macrophages, indicating that the threshold for inflammasome activation is variable throughout granulopoiesis and different cell lines. The work of Bauernfeind et al. (2012) has shed light on a key mechanism in the inflammatory cascade, a process implicated in numerous diseases. Finding endogenous and exogenous mediators that can control the unchecked inflammation seen in these conditions is an important step to discover pharmaceutical intervention and even possible cures. Hopefully these studies will set the foundation for the exploration of the regulatory effects of miR-223 and other miRNAs in regulating inflammation in numerous medical conditions in the near future.
